# Efficacy and safety of a new elastic tourniquet cuff in total knee arthroplasty: a prospective randomized controlled study

**DOI:** 10.1186/s12938-017-0393-0

**Published:** 2017-08-08

**Authors:** O-Sung Lee, Myung Chul Lee, Hyuk-Soo Han

**Affiliations:** 1 Department of Orthopaedic Surgery, Seoul National University College of Medicine, Bundang Hospital, 166 Gumi-ro, Bundang-gu, Seongnam, Gyeonggi-do 463-707 South Korea; 2 Department of Orthopedic Surgery, Seoul National University Hospital, 101 Daehak-ro, Jongno-gu, Seoul, 110-744 South Korea

**Keywords:** Total knee arthroplasty, Tourniquet, Automatic pneumatic tourniquet, Cuff

## Abstract

**Background:**

The effects of cuff shape, timing of tourniquet application, and automated systems using limb occlusion pressure (LOP) have been reported to minimize the appropriate tourniquet pressure. However, studies on the raw material of the cuff itself to reduce the complications related to the tourniquet have been very rarely reported. The purpose of this study is to report the efficacy and safety of a tourniquet system with a new elastic cuff in which pressure is set with LOP in total knee arthroplasty (TKA).

**Methods:**

A total of 63 patients who underwent primary TKA for osteoarthritis were enrolled from July to December 2015. Thirty-one patients were allocated to the new elastic cuff group and 32 in the conventional cuff group. Bloodless surgical field, pain visual analog scale (VAS) on the thigh, thigh circumference, range of motion, incidence of deep vein thrombosis, and muscle enzyme level after surgery were checked and compared between the 2 groups.

**Results:**

Only 1 of the 31 patients in the elastic cuff group required more pressure for obtaining a bloodless surgical field, whereas 4 of the 32 patients in the conventional cuff group required more pressure to complete surgery without being disturbed by sustained bleeding. Two patients in the conventional cuff group needed treatment for blisters and bullae at the tourniquet application site. There was no difference in pain VAS score, thigh circumference, range of motion, incidence of deep vein thrombosis, and level of muscle enzyme.

**Conclusions:**

A new elastic tourniquet cuff provided a more proper bloodless surgical field with less adjustment of tourniquet pressure despite a similar level of tourniquet pressure compared to the conventional cuff and had a low incidence of skin complications on the site of tourniquet application in TKA. These benefits make it an effective and safe medical device for orthopedic surgery requiring a tourniquet, such as TKA.

## Background

The pneumatic tourniquet is an efficient device to achieve a bloodless surgical field in orthopedic surgeries. It is a well-known method to provide such bloodless surgical field, facilitate cementation, and reduce the duration of surgery, especially in primary total knee arthroplasties (TKAs). However, the conventional tourniquet pressure in TKAs was known to be between 300 and 350 mmHg, and such a high inflation pressure has risks of complications, including ischemic pain, swelling, skin problem, rhabdomyolysis, deep vein thrombosis (DVT), and nerve palsy [1–3]. Thus, it is recommended to reduce tourniquet pressure to minimize potential risks of tourniquet complications [4].

Over a long period, many studies have reported the effect of the shape of the cuff, timing of tourniquet application, and automated tourniquet system to minimize the proper tourniquet pressure [5–7]. As one of the effective methods to reduce tourniquet pressure to occlude blood flow distal to the tourniquet application site, tourniquet systems using minimal limb occlusion pressure (LOP) were introduced [7, 8]. Recently, LOP is detected automatically by blood flow transducers with gradual increments of tourniquet pressure until blood flow distal to the site of cuff application is interrupted [9]. It has been well known that tourniquet inflation pressures with these LOP systems were generally lower than the inflation pressures based on the surgeon’s experiences [10].

**Fig. 1:**
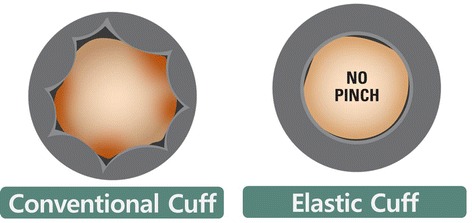
Comparison of the inner surfaces of the inflated conventional cuff and elastic cuff

However, LOP could be influenced by patient’s age, systolic blood pressure, materials of the cuff, width and length of the cuff, and limb circumference [11, 12]. Especially, standard cuffs were not flexible enough to cover the entire circumference of the limbs owing to the folds on the inner side of the cuff while the tourniquet inflates (Fig. 1). These folds can cause pinching of the superficial skin, postoperative pain, and leakage of blood flow that could disturb the bloodless surgical field. Thus, adjusting the materials of the cuff as well as the tourniquet pressure to maximize the effectiveness and safety of this tourniquet system based on LOP seems necessary. However, it is possible to concern the risk that blood flow could be easily passed during manipulation of extremities on surgery because of its flexible and elastic nature. Therefore, the hypotheses of this study were (1) a new elastic cuff would result in better bloodless surgical field without the folds on the inner side of cuff than conventional cuff, (2) it would be safe and effective enough to be applied in TKAs despite of its elasticity.

## Methods

### Automated pneumatic tourniquet system based on the LOP and elastic cuff

**Fig. 2:**
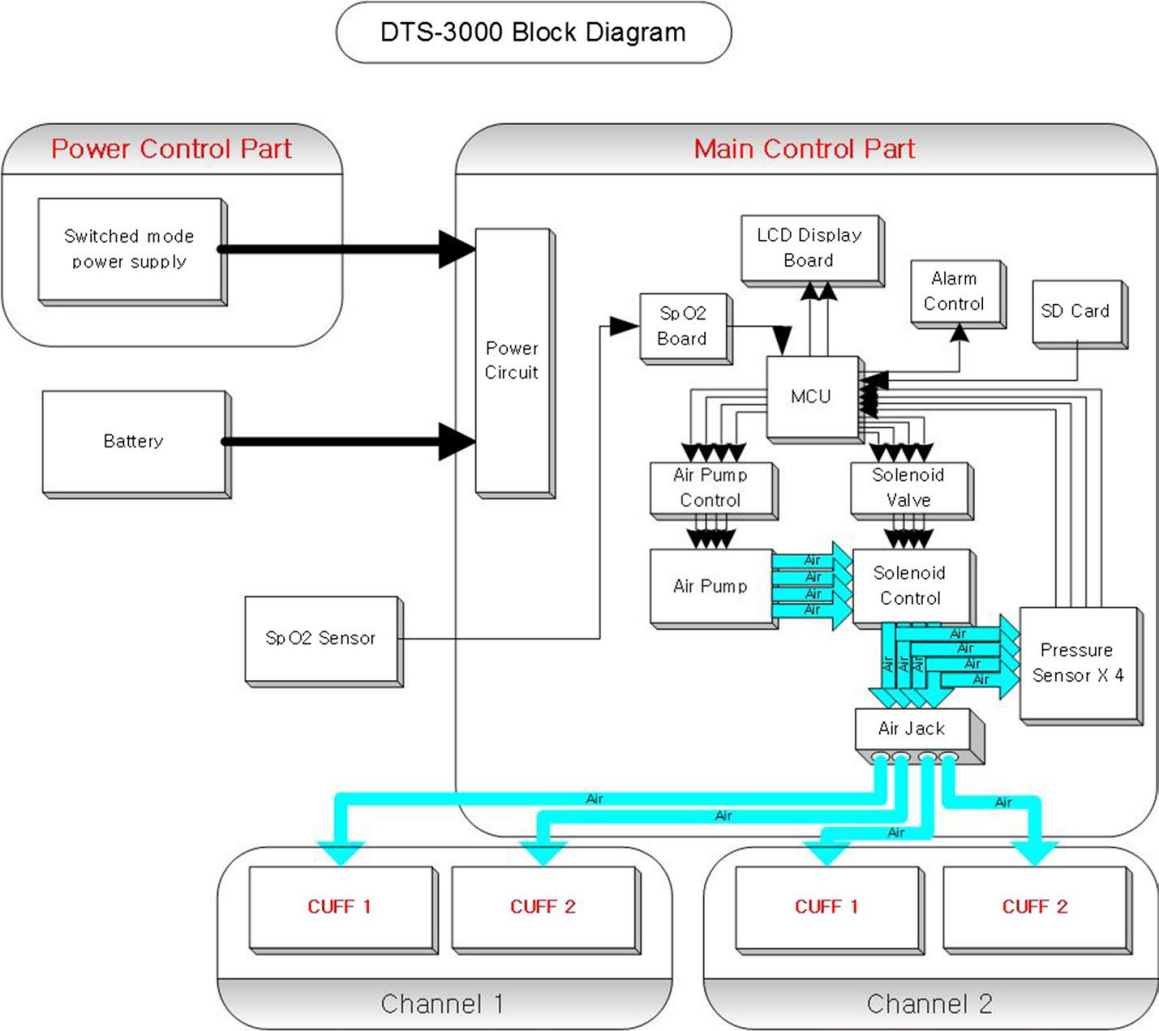
Block diagram of automated pneumatic tourniquet based on LOP (DTS-3000). The automated pneumatic tourniquet system contains a blood pressure monitoring module, pulse oximetry sensor, an air pump system and a pressure sensor

The automated pneumatic tourniquet system (DTS-3000, DSMAREF, KOR), which was used in this trial, consist of main control part and power control part (Fig. 2). The main control part consists of a microcontroller, an air pump, a solenoid, and a pressure sensor. The setup of LOP was initiated by placing a pulse oximetry sensor on a digit of the surgical site. The cuff automatically inflates to the pressure that the patient’s pulse signal is not detected and then deflates while the patient’s pulse signal is detected and the sensor parameters are adjusted automatically. The recommended tourniquet pressure (RTP) based on LOP is automatically set through the algorithm, which the users can set a safety margin according to the LOP. After that, the results of the LOP and the RTP are shown on the display panel. And then, the pump runs and the solenoid valve opens. When the pressure sensor detects the targeted RTP, the solenoid valve closes and the pump stops. The sensor continuously monitors the pressure and maintains the target pressure using the solenoid and the pump. The clinician can manually adjust the tourniquet pressure at any time.

The elastic cuff is made of silicone rubber as the main material, while the conventional cuff is made of polyurethane elastomers. The width and the length of all cuffs were 12 and 90 cm, respectively. When the cuff inflates, the length of the inside circumference and the outside circumference must be different to minimize the folds on inner side of cuff (Fig. 1). To get rid of the pinch due to these folds, the outer surface should be stretched and the inner surface should be shrunk. Therefore, the elastic cuffs were made of silicone rubber of which hardness decreased from 90 ± 3 shore A to 57 shore A, and elastic modulus increased from 450 to 785% (Table 1).

**Table 1:** Comparison between the conventional cuff and elastic cuff

	Conventional cuff	Elastic cuff
Raw material	Thermoplastic polyurethane elastomer	Silicone rubber
Sterilization method	Ethylene oxide gas	Autoclave
Hardness	90 ± 3, shore A	57, shore A
Tensile strength (MPa)	39.2	10.6
Elongation (%)	450	785
Tear strength (kg/cm)	>100	52

### Volunteer study prior to the prospective, double-blinded randomized controlled trial

Prior to the prospective, double-blinded randomized controlled trial, a clinical study with 20 healthy volunteers was conducted to test the safety of the elastic cuff and automated pneumatic tourniquet system. The elastic cuff was applied to one thigh, and the conventional cuff was applied to the other thigh to occlude blood flow to the lower extremities according to a random-number table. As a result of the volunteer study, the mean pain visual analog scale (VAS) score after inflation of the tourniquet for 5 min was lower with the elastic cuffs than with the conventional cuffs (3.30 ± 1.60 vs. 4.08 ± 1.72, *p* = 0.04). LOP checked on the index finger and second toe using a blood flow transducer was not significantly different between the two cuffs (elastic cuff: 143.6 ± 21.7 mmHg, conventional cuff: 147.3 ± 22.8 mmHg, *p* = 0.566). However, LOP evaluated using duplex ultrasonography on the radial artery and posterior tibial artery distal to the tourniquet application site was lower with the elastic cuff (153.4 ± 21.0 mmHg vs. 162.3 ± 17.4 mmHg, *p* = 0.057), and the LOP not allowing a leakage after 10 times of passive full range of motion (ROM) of the knee was statistically significantly lower with the elastic cuff than with the conventional cuff (156.6 ± 19.8 mmHg vs. 163.8 ± 17.8 mmHg, *p* = 0.033) (Table 2). Further, age, body mass index, and circumference of the extremity had no influence on the results of pain VAS and LOPs using the linear mixed-effects model (data not shown).

**Table 2:** Summary of the volunteer study

	Conventional cuff group	Elastic cuff group	*p* value
Pain VAS	4.1 ± 1.7	3.3 ± 1.6	0.04
LOP (mmHg)	147.3 ± 22.8	143.6 ± 21.7	0.566
LOP (D) (mmHg)	162.3 ± 17.4	153.4 ± 21.0	0.057
LOP (ROM) (mmHg)	163.8 ± 17.8	156.6 ± 19.8	0.033

*VAS* visual analog scale, *LOP* pressure checked with a blood flow transducer on the index finger and second toe, *LOP (D)* pressure checked with a duplex ultrasonography on the radial artery and posterior tibial artery, *LOP (ROM)* pressure not allowing a leakage after 10 times of passive full range of motion on the elbow and knee with a duplex ultrasonography on the radial artery or posterior tibial artery

### Patients

Following this volunteer study, a prospective, double-blinded randomized controlled trial enrolled 68 patients who underwent primary TKA for osteoarthritis between July and December 2015. Both groups were treated identically in all respects except for the kind of cuff being tested, and patients and observers were blinded to which group an individual was assigned. Patients with primary osteoarthritis between 50 and 80 years old scheduled for primary TKA were included and randomly allocated to the elastic cuff and conventional cuff groups. Patients were excluded if they had a history of anticoagulation therapy, bleeding disorder, DVT, psychiatric illness, active malignancy, chronic alcoholism, or surgery of the extremities. Further, duplex ultrasonography was conducted to identify preoperative DVT in all subjects. Three of the 68 patients required switching to general anesthesia because of the failure of spinal anesthesia, and blood flow using the transducer was not detected in 2 patients because of nail opacity due to scarring and permanent dystrophy of the nails. Finally, 31 patients were allocated in the elastic cuff group and 32 patients in the conventional cuff group. The demographics showed no significant difference between the two groups (Table 3).

**Table 3:** Demographics

	Conventional cuff group	Elastic cuff group	*p* value
Patients (n)	31	32	
Sex (male:female)	3:18	3:19	
Age (year)	69.1 ± 5.3	68.1 ± 5.9	0.597
Body weight (kg)	65.8 ± 9.1	64.0 ± 11.4	0.576
Height (cm)	156.1 ± 6.1	155.8 ± 6.3	0.900
Body mass index (kg/m^2^)	27.2 ± 4.5	26.2 ± 3.4	0.450
Thigh circumference (cm)	49.9 ± 4.6	50.4 ± 4.5	0.528
SBP (mmHg)	132.1 ± 17.7	129.0 ± 19.3	0.593

Values are presented as mean ± standard deviation and derived using the paired t test. *SBP* systolic blood pressure

### Strategy of tourniquet application and surgical techniques

After the anesthesiologist finished the spinal anesthesia induction and blood pressure was stable, we applied the RTP automatically based on the LOP, which was checked with a blood flow transducer on the second toe. RTP was set at the automatically measured LOP plus a safety margin using the modified McEwen’s guideline [13]. The safety margin was defined as 50 mmHg for LOPs of <130, 75 mmHg for those between 110 and 215, and 100 mmHg for those of >215 mmHg. The skin under the tourniquet was covered with a single layer of cast padding on a single layer of stockinette. The operated leg was elevated and exsanguinated with an Esmark bandage before inflating the automatic pneumatic tourniquet. The tourniquet was inflated just before the skin incision and deflated after the cementation was completed. The primary TKAs were performed using the same technique in both groups. After a midline skin incision, a medial parapatellar approach was performed. Intramedullary guide was used for the femur and extramedullary guide for the tibia. The posterior cruciate sacrificed and fixed-bearing TKA were implanted in all patients. All prostheses were fixed with cement. An intra-articular closed-suction drainage after TKA was used for 24 h in both groups.

### Evaluation

The primary outcome was postoperative pain levels of the thigh where the cuff was applied on. The LOP and RTP during the TKAs were recorded. Additionally, surgeons evaluated the bloodless surgical field. If surgeons needed to raise the tourniquet pressure due to excessive bleeding on the operative field, we defined the case as a failure to obtain a bloodless surgical field. Pain VAS score, ROM, and thigh circumference on the preoperative day of surgery and postoperative days 1, 2, 4, and 7 were evaluated. The values of creatine phosphokinase (CPK) and lactate dehydrogenase (LDH) at postoperative days 1, 2, 4, and 7 were used as parameters of muscle injury. The number of rescue injections during the total hospital stay was counted. The duration and number of patient-controlled analgesia (PCA) refill were also counted. We inspected the incidence of skin complication that required treatment and conducted duplex ultrasonography for postoperative DVT evaluation at postoperative day 7. Postoperative blood loss was also recorded as the total closed-suction drainage output for 24 h after TKA.

### Statistical analysis

All statistical analyses were performed with the SPSS version 22.0 (SPSS Corp., Chicago, USA). Data description was based on means and standard deviations (SDs) for continuous values. Comparisons of continuous variables between two groups were accomplished using the Student’s *t* test and differences of other categorical variables between the two groups were analyzed with Chi square test and Fisher’s exact test. And the differences during the postoperative follow-up period in both groups were detected using ANOVA. A *p* value <0.05 was considered significant.

## Results

**Fig. 3:**
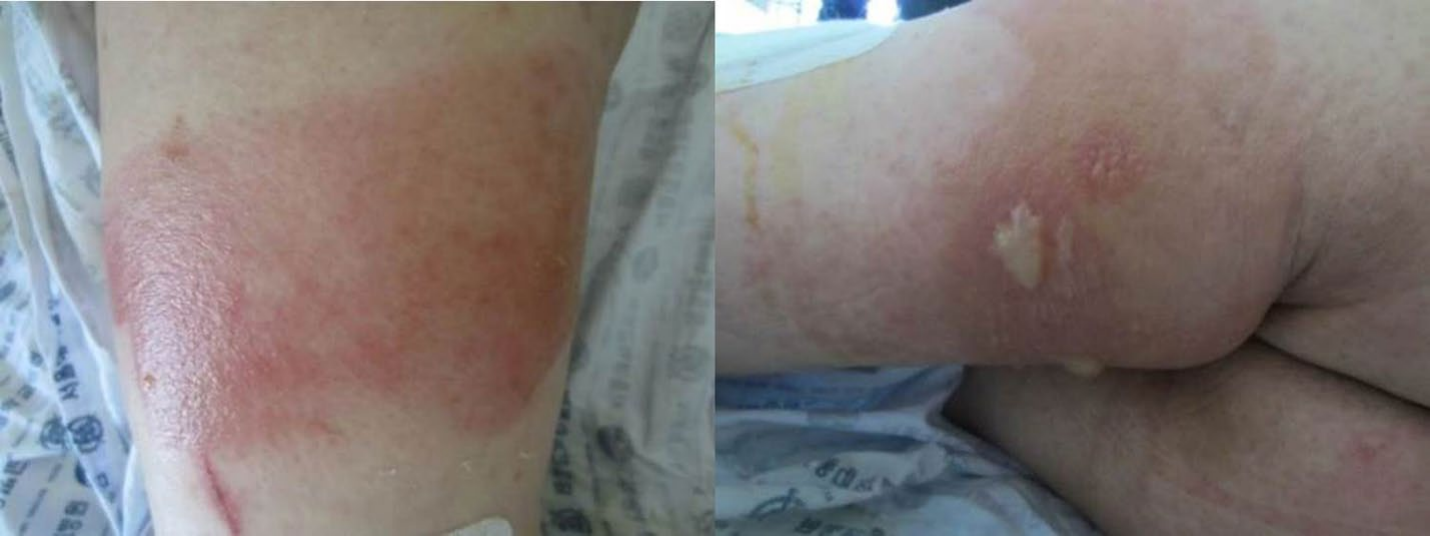
A 64-year-old male patient presenting severe bullous skin eruption on the site of conventional cuff application

The average value of LOP in the elastic cuff group was lower than that in the conventional cuff group with no statistical difference (175.7 ± 18.8 vs. 169.3 ± 20.4, *p* = 0.294). The average RTP was also lower in the elastic cuff group with no statistical difference (252.3 ± 21.0 vs. 246.1 ± 23.7, *p* = 0.374). In terms of bloodless surgical field, the surgeon had to raise the tourniquet pressure to achieve a bloodless surgical field in 4 cases in the conventional cuff group, on the other hand, there was only 1 case of unacceptable bloodless surgical field in the elastic cuff group [odds ratio (OR) 4.59, 95% confidence interval (CI) 0.48–43.63, *p*  =  0.196]. In terms of DVT, no patient had DVT before surgery, however, small isolated distal DVTs were found in 6 out of the 31 patients in the conventional cuff group and 6 out of the 32 patients in the elastic cuff group on the duplex ultrasonography at postoperative day 7 (OR 1.04, 95% CI 0.296–3.66, *p*  =  0.951). However, all DVTs were asymptomatic and found in the small muscular branch of the calf, which does not require an anticoagulant therapy. Postoperative blood loss for 24 h after TKA, duration and number of PCA refill, and number of rescue injections showed no statistically significant difference in both groups (Table 4). There was also no significant difference in pain VAS score, thigh circumference, and ROM at each period between the two groups. In terms of muscle damage, the levels of CPK and LDH at each period showed little difference except for the level of CPK on postoperative day 2 (Table 5). Two cases in the elastic cuff group presented severe bullous skin eruption at the site of tourniquet application that required skin treatments, such as dressings and wound care until his discharge date (Fig. 3).

**Table 4:** Comparison of the intraoperative and postoperative outcomes

	Conventional cuff group	Elastic cuff group	*p* value
LOP (mmHg)	175.7 ± 18.8	169.3 ± 20.4	0.294*
RTP (mmHg)	252.3 ± 21.0	246.1 ± 23.7	0.374*
Tourniquet time (min)	67.3 ± 18.4	62.9 ± 17.0	0.412*
Drainage for 24 h after TKA	563.2 ± 262.8	616.6 ± 327.1	0.559*
Duration of PCA days	4.57 ± 1.43	4.64 ± 1.40	0.881*
Refills of PCA (n)	1.19 ± 0.40	1.05 ± 0.21	0.145*
Rescue injections (n)	1.29 ± 1.45	1.00 ± 1.27	0.496*
Unacceptable bloodless surgical field (n)	4	1	0.196^†^
Skin complication	2	0	0.238^†^
Acute DVT on duplex ultrasonography (n)	6	6	0.951^§^

Values are presented as mean ± standard deviation (*Student’s t test; ^†^Fisher’s exact test; ^§^Chi square test)

*LOP* limb occlusion pressure, *RTP* recommended tourniquet pressure, *TKA* total knee arthroplasty, *PCA* patient-controlled anesthesia, *DVT* deep vein thrombosis

**Table 5:** Comparison of the pain VAS score, thigh circumference, ROM, CPK, and LDH with time table

	Preoperative	Postoperative day 1	Postoperative day 2	Postoperative day 4	Postoperative day 7
Pain VAS on the thigh					
Conventional cuff		1.3 ± 2.3	1.3 ± 2.8	0.6 ± 1.4	0.3 ± 0.8
Elastic cuff		1.3 ± 1.9	0.5 ± 1.2	0.2 ± 0.7	0.1 ± 0.4
*p* value		0.960	0.230	0.308	0.421
Thigh circumference (cm)					
Conventional cuff	49.9 ± 4.6	52.0 ± 4.5	51.9 ± 5.0	51.0 ± 4.6	50.4 ± 4.3
Elastic cuff	50.4 ± 4.5	52.0 ± 4.5	51.7 ± 4.5	50.9 ± 4.2	50.6 ± 4.2
*p* value	0.528	0.992	0.865	0.901	0.849
ROM (°)					
Conventional cuff	116.9 ± 21.2	55.5 ± 14.3	84.0 ± 18.3	107.1 ± 15.5	124.8 ± 11.8
Elastic cuff	125.0 ± 18.6	53.9 ± 13.9	77.5 ± 18.0	104.3 ± 17.5	121.8 ± 13.5
*p* value	0.190	0.710	0.244	0.579	0.451
CPK (mcg/L)					
Conventional cuff		94.4 ± 35.6	110.0 ± 28.4	76.1 ± 35.3	58.7 ± 33.2
Elastic cuff		134.8 ± 94.2	163.2 ± 110.0	106.2 ± 73.2	63.7 ± 34.9
*p* value		0.072	0.038	0.096	0.632
LDH (IU/L)					
Conventional cuff		178.8 ± 34.9	175.9 ± 35.8	201.2 ± 33.8	230.5 ± 63.0
Elastic cuff		184.1 ± 35.8	188.5 ± 36.0	211.2 ± 41.3	232.9 ± 33.5
*p* value		0.624	0.257	0.394	0.874

Values are presented as mean ± standard deviation and derived using ANOVA

*VAS* visual analog scale, *ROM* range of motion, *CPK* creatine phosphokinase, *LDH* lactate dehydrogenase

## Discussion

The most important finding of the present study is that the elastic cuff with automated pneumatic tourniquet system based on LOP can more effectively occlude blood flow with less adjustment of tourniquet pressure to achieve a bloodless surgical field despite a similar level of RTP compared with the conventional cuff in TKA. Further, in terms of pain, incidence of DVT and skin complications, the elastic cuff is safe enough to be applied in TKA despite of its elasticity.

Pneumatic tourniquets are very common and useful equipment to achieve a bloodless surgical field in the surgeries of the extremities. However, a higher inflation pressure has a higher risk of complications, such as sustained thigh pain, neurologic deficit, compartment syndrome, and even systemic complications, such as hypertension and DVT [1, 2, 4, 13–15]. Recent systematic reviews recommended to apply tourniquet for <2 h and deflate it for short intervals of 10–15 min to minimize the risks of complications related to tourniquet application [16, 17]. Considering such a limited time, it is important to decrease the tourniquet pressure to prevent complications associated with tourniquet application. It is usually recommended to set the tourniquet cuff pressure based on the systolic blood pressure plus 50–100 mmHg for the upper extremity and double systolic blood pressure for the lower extremity (or 200–250 mmHg for the upper extremity and 250–350 mmHg for the lower extremity) according to the Modification of the Bruner’s Ten Rules [18]. Many surgeons still apply tourniquet with a typical pressure of 300–350 mmHg especially in cases of TKA [1, 14, 19]. To decrease the tourniquet pressure, many studies suggested the LOP as the minimum pressure required to stop the blood flow distal to the cuff [12, 16, 20]. However, LOP essentially depends on various factors. The factors related to the patients are systolic blood pressure, limb circumference, and tissue conditions, and those related to the tourniquet system are the width and shape of the cuff and the method of cuff application. McEwen et al. [8] presented the use of the automated LOP, which decreased the average thigh tourniquet pressures by 19–42% from the typical 300–350 mmHg. In terms of cuff design, Younger et al. [7] reported that a wide-contoured cuff allowed a lower tourniquet pressure. They reported that the standard cuff maintained an acceptable bloodless field for 18 of 20 patients at an average pressure of 242 mmHg, and the wide cuff was acceptable for 19 of 20 patients at an average of 202 mmHg.

Even though there have been many research conducted on the design and pressure associated with tourniquets, only a few reported on cuff material. Especially, there has been no research with the elastic cuff made of silicon rubber. The conventional cuff is usually made of polyurethane elastomers, which make stiff folds on the inner surface of the cuff when inflated. These stiff folds result in pinches that cause not only skin complications but also sustained pain (Fig. 1). Moreover, if the folds on the inner side of the cuff become larger, it can cause blood flow leakage distal to the cuff. This effect is disadvantageous in providing an even pressure under the tourniquet application site. Thus, our new elastic cuff made of silicone rubber was more flexible, could reduce patient’s pain and skin complications, and provide an even pressure while preventing a blood flow leakage. Contrary, it can be predicted that blood flow could easily pass across the cuff during manipulation of the extremities on the operations due to its flexible and elastic nature, especially, if a surgeon apply a relatively low pressure in the LOP system. However, our two trials, with healthy volunteers and subsequent TKA patients, presented that elastic cuffs were not inferior to conventional cuffs even at low pressures in the LOP system despite concerns about its elastic property.

In our volunteer study, the elastic cuff was superior to the conventional cuff in terms of pain, and the pressures at the same conditions of measurement were lower with the elastic cuffs. The pressures checked in the radial and posterior tibial arteries proximal to the digit were lower with the elastic cuffs. These findings indicate that elastic cuffs have a greater possibility to secure a bloodless surgical field without blood flow leakage by applying a lower pressure to block the blood flow to the actual surgical site. The tourniquet pressure after the ROM exercises was also lower with the elastic cuffs. This result presented that the tourniquet pressure to avoid the leakage of blood flow after limb manipulation was lower with the elastic cuff. A lower tourniquet pressure can be expected to result in fewer complications, such as pain, swelling, and skin problems. In this randomized study on TKA patients, the LOP and RTP were lower in the elastic cuff group although there was no statistical significance. There was also no significant difference in the pain VAS scores, short-term recovery of knee function, swelling, and muscle enzyme. However, when RTP based on individually measured LOP applied to each patient, there were more cases of unacceptable surgical viewing in the conventional cuff group than in the elastic cuff group. Furthermore, there was no skin complication in the elastic cuff group. If there had been a control group of TKA patient using a typical tourniquet pressure more than 300 mmHg, the difference would have been more obvious.

Although there was no control group using typical tourniquet pressure of 300 mmHg in this study, our tourniquet application strategy based on the LOP system and elastic cuff has some benefits in terms of safety and efficacy compared to conventional application of tourniquet during TKA in previous studies [3, 16, 21, 22]. First, our tourniquet pressure based on the LOP system and elastic cuff (246.1 ± 23.7 mmHg) was markedly lower than that with conventional application of tourniquet during TKA in previous studies (275–350 mmHg) [3, 16, 22]. Second, it showed the lower incidence of complications related to the tourniquet. Watanabe et al. [3] reported that 19 cases of venous thromboembolism occurred after 42 TKAs with a conventional cuff, compared with 6 out of 32 TKAs in our elastic cuff group. Heller et al. [22] reported that 18 skin blisters were observed after 335 TKAs (5.4%), compared with none of the elastic cuff group in our study. Finally, the LOP system and elastic cuff effectively provided a bloodless surgical field during TKA. Olivecrona et al. [21] reported an average of 8.7 points for bloodless field during TKA when applying mean 246 ± 45 mmHg based on the LOP with conventional cuff (1 point indicating the worst and 10 points indicating the most optimal). Although the bloodless surgical fields were not scored in our study, the elastic cuff could be considered effective enough to achieve the bloodless surgical field during TKA because only one out of 32 patients needed more pressure despite an average tourniquet pressure (246.1 ± 23.7 mmHg) similar to that of Olevecrona’s report [21].

Some limitations of this study should be mentioned. First, although we confirmed that there was no significant difference in the demographics between the two groups, the patients may have uncontrolled factors, such as unrevealed atherosclerosis that can affect the LOP and efficacy of the tourniquet. However, since we conducted a prospective randomized controlled study, which has strictly followed the inclusion and exclusion criteria, the selection bias was expected to be minimal. Second, the evaluation of the bloodless surgical field can be criticized as too subjective depending on the operator. Thus, instead of using a subjective scoring system to evaluate bloodless surgical field, we counted the number of cases in which the operator who was blinded to the randomization had to increase the tourniquet pressure to achieve a bloodless surgical field. Third, the differences in pain and complications did not prominently be noticed depending on the cuff material, because relatively low pressures of 250 mmHg set by the LOP system were equally applied to both group. However, the purpose of this paper is not to demonstrate the superiority of elastic cuff over conventional cuff, but to demonstrate that the elastic cuff is efficient and safe for clinical use in TKA surgery in terms of postoperative skin complications and achieving a bloodless surgical field, which was confirmed in this study.

## Conclusion

In this prospective randomized clinical study, the elastic cuff with a LOP system was efficient in achieving a bloodless surgical field and safe enough to be applied to TKA patients in terms of postoperative skin complications related to tourniquet system. There was no statistically significant difference in pain VAS score, ROM, thigh circumference, muscle enzyme level, postoperative blood loss, duration and number of PCA refill, and number of rescue injections in both groups at the early postoperative period after TKA. The elastic cuffs could be an effective and safe medical device for orthopedic surgeries requiring a tourniquet, such as TKAs without any other risk caused by its elasticity.

## Abbreviations

LOP: limb occlusion pressure
RTP: recommended tourniquet pressure
DVT: deep vein thrombosis
TKA: total knee arthroplasty
ROM: range of motion
VAS: visual analog scale
CPK: creatine phosphokinase lactate dehydrogenase
LDH: lactate dehydrogenase
